# The role of astrocytes in CNS tumors: pre-clinical models and novel imaging approaches

**DOI:** 10.3389/fncel.2013.00040

**Published:** 2013-04-16

**Authors:** Emma R. O'Brien, Clare Howarth, Nicola R. Sibson

**Affiliations:** Department of Oncology, CR-UK/MRC Gray Institute for Radiation Oncology and Biology, Churchill Hospital, University of OxfordOxford, UK

**Keywords:** astrogliosis, brain metastases, glioma, nuclear imaging, MRI

## Abstract

Brain metastasis is a significant clinical problem, yet the mechanisms governing tumor cell extravasation across the blood-brain barrier (BBB) and CNS colonization are unclear. Astrocytes are increasingly implicated in the pathogenesis of brain metastasis but *in vitro* work suggests both tumoricidal and tumor-promoting roles for astrocyte-derived molecules. Also, the involvement of astrogliosis in primary brain tumor progression is under much investigation. However, translation of *in vitro* findings into *in vivo* and clinical settings has not been realized. Increasingly sophisticated resources, such as transgenic models and imaging technologies aimed at astrocyte-specific markers, will enable better characterization of astrocyte function in CNS tumors. Techniques such as bioluminescence and *in vivo* fluorescent cell labeling have potential for understanding the real-time responses of astrocytes to tumor burden. Transgenic models targeting signaling pathways involved in the astrocytic response also hold great promise, allowing translation of *in vitro* mechanistic findings into pre-clinical models. The challenging nature of *in vivo* CNS work has slowed progress in this area. Nonetheless, there has been a surge of interest in generating pre-clinical models, yielding insights into cell extravasation across the BBB, as well as immune cell recruitment to the parenchyma. While the function of astrocytes in the tumor microenvironment is still unknown, the relationship between astrogliosis and tumor growth is evident. Here, we review the role of astrogliosis in both primary and secondary brain tumors and outline the potential for the use of novel imaging modalities in research and clinical settings. These imaging approaches have the potential to enhance our understanding of the local host response to tumor progression in the brain, as well as providing new, more sensitive diagnostic imaging methods.

Metastasis, the spread of cancer from the primary tumor site to distant organs, is the leading cause of cancer morbidity and mortality and 10–40% of all cancer patients will develop metastatic spread to the brain (Nussbaum et al., 1996). However, our understanding of brain metastasis is still incomplete, and the unique microenvironment of the CNS, both on a cellular and metabolic basis, means mechanistic insights from peripheral organs cannot be readily translated. The processes underlying the extravasation of neoplastic cells across the blood-brain barrier (BBB), their subsequent colonization of the perivascular space (Carbonell et al., 2009) and later the parenchyma, are yet to be fully characterized. Progress in this area is limited by the lack of robust *in vitro* assays that truly reflect the complex nature of the CNS. It is necessary, therefore, to develop better pre-clinical models, in tandem with sophisticated imaging modalities, to better allow the investigation of the pathogenic mechanisms underlying metastasis progression. In turn, imaging may identify novel biomarkers for early tumor detection and new therapeutic avenues.

Astrocytes are the most abundant member of the glial family and have multiple roles in the central nervous system. As well as providing structural support for neurons and the BBB, as outlined below, they play an integral role in maintaining CNS function, participating in synaptic activity, mediating ionic and transmitter homeostasis, and regulating blood flow. At the same time, astrocytes actively respond to challenges such as infection, injury, ischemia, and neurodegeneration, by changing their transcriptional profile and morphology in the process of reactive astrogliosis, which has been extensively reviewed (Pekny and Nilsson, 2005; Sofroniew, 2009; Middeldorp and Hol, 2011) and is characterized by up-regulation of glial fibrillary acidic protein (GFAP).

This review aims to detail the role of astrocytes in both primary and secondary CNS tumors, as determined from both *in vitro* studies and *in vivo* pre-clinical models. Secondly, novel imaging techniques, many of which have been successfully used in other neuropathologies, will be discussed in the context of investigating astrogliosis in CNS tumors, both *in vivo* and potentially in the clinic.

## Pre-clinical models; mechanistic insights into the role of astrocytes in CNS tumors

### Primary brain tumors

Besides the malignant astrocytes that comprise many primary brain tumor sub-types, a role for *stromal* astrocytes in tumor progression has been constructed through human biopsy samples, co-culture *in vitro* experiments and transgenic *in vivo* models. Broadly speaking, two main forms of malignant brain tumor exist, as classified according to cellular origin; oligodendrocytomas, comprised of oligodendrocytes, and astrocytic neoplasms, also known as gliomas, which can be further stratified into diffuse astrocytoma, anaplastic astrocytoma, and glioblastoma multiforme (GBM) (Louis et al., 2007). GBM consists of neoplastic astrocytes that are poorly differentiated, and is characterized by high invasive potential and angiogenesis. It is the most commonly diagnosed and aggressive glioma in adults and, hence, will be the focus of work described here.

**Figure 1:**
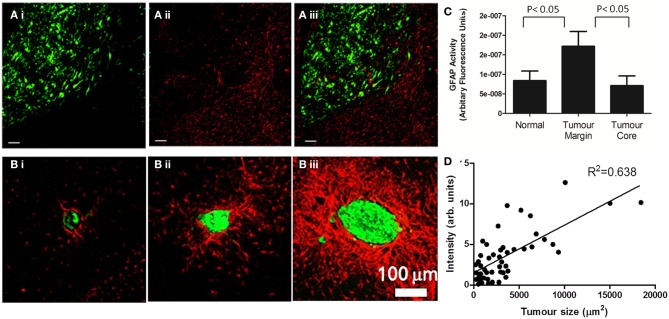
**Astrocyte activation, as determined by GFAP staining, is present in the peri-tumoral area of both primary (A) and secondary (B) tumors. (A)** (i) In a mouse model of glioma, the growth of DBRTG glioma cells can be seen in green (GFP labeled), adjacent to a wall of astrocyte activation, seen in red (Alexa555 probe) (ii). The merged image (iii) indicates little infiltration of activated astrocytes into the tumor mass, as quantified by arbitrary fluorescence units of GFAP activity **(C)**. Figure adapted from Lee et al. (2011). **(B)** In a mouse model of lung derived brain metastasis, in which HARA-B cells were inoculated intra-cardially, astrocyte activation (Cy3 probe, red) was observed surrounding metastatic growth (Alexa488 probegreen). As with the glioma model, astrocytes are present in the tumor periphery, rather than the core. The extent of astrocyte activation increased with tumor size **(D)**. Figure adapted from Seike et al. (2011).

Immunohistochemistry reveals reactive astrocytes surrounding glioma in both human biopsies (Nagashima et al., 2002) and murine models, as shown in Figure 1, and have been proposed to have pro-immunogenic roles. For instance, glioma associated astrocytes have a markedly different mRNA expression profile to normal astrocytes, primarily displaying components of the antigen presentation pathway, such as MHC Class II proteins (Katz et al., 2012). This observation suggests that astrocytes interact with “helper” T cells, leading to localized inflammation. Astrocytes surrounding human glioma biopsies secrete CCL2, a macrophage and T cell recruiting chemokine, with a strong positive correlation between CCL2 expression and T cell infiltration (Carrillo-De Sauvage et al., 2012). However, as with all aspects of the immune response to tumor growth, it does not necessarily follow that the presence of T cells leads to tumor cell clearance. Indeed, Barcia et al. demonstrate that although T cells infiltrate the GBM microenvironment, very few cytotoxic T cells (CTLs) make immunological synapses with tumor cells, whilst there is a greater population of potential regulatory T cells (Barcia et al., 2009), which attenuate immune responses.

Further evidence that this interaction downregulates immune function comes from co-culture experiments. Co-culture of either GBM astrocytes or normal human astrocytes with T cells, leads to a downregulation of IFN-γ production (Kostianovsky et al., 2008), hence inhibiting effector function. Additionally, astrocytes have been shown to directly induce T cell apoptosis via cell-cell contacts; astrocytoma derived astrocytes express Fas ligand (FasL) which interacts with Fas expressing cells, such as T cells, to induce cytolysis (Saas et al., 1997). This mechanism is not restricted to malignant astrocytes, but has been demonstrated in normal astrocytes (Bechmann et al., 1999, 2002), suggesting that astrocytes in the glioma periphery could also be involved in repressing anti-tumor immune function.

Interactions with microglia, as well as infiltrating monocytes, also indicate that astrocytes could play a role in modulating immune function in the tumor periphery. Both GBM derived malignant astrocytes and normal human astrocytes suppressed TNF secretion by microglia and monocytes, and also inhibited the ability of these cell types to activate T cells owing to downregulation of co-stimulatory molecules (Kostianovsky et al., 2008).

Interleukin-1β (IL-1β) has been identified in reactive astrocytes in the glioma periphery, suggesting an anti-tumor immune response (Nagashima et al., 2002). Conversely, *in vitro*, IL-1β has been implicated in inducing glioma invasion (Bryan et al., 2008; Huang et al., 2009), as well as promoting tumor growth via autocrine induction of TNF signaling (Chung and Benveniste, 1990; Adachi et al., 1992). Other astrocyte-derived cytokines, such as stromal derived factor-1(SDF-1), have also been identified *in vitro* as enhancing glioma growth (Barbero et al., 2003) and invasiveness (Zhang et al., 2005). Furthermore, this invasive phenotype has been demonstrated *in vivo* using a transgenic mouse model in which glioma is induced by platelet derived growth factor (PDGF) over-expression. In this study, stromal astrocytes in the peri-tumoral region were shown to express elevated levels of Tenascin-C (TN-C) (Katz et al., 2012), an extra-cellular matrix glycoprotein, previously shown to promote GBM invasion (Sarkar et al., 2006).

Matrix metalloproteinases (MMPs) have been proposed to play a key role in mediating glioma invasion through the parenchyma, and peri-tumoral reactive astrocytes have been shown, immunohistochemically, to express MMP2 in post-mortem samples (Nagashima et al., 2002). At the same time, *in vitro* co-culture experiments between human fetal astrocytes and human glioma cell lines (U251N and U87), indicate that soluble factors released by glioma cells cleave the MMP2 pro-enzyme to its active form (Le et al., 2003), demonstrating the dynamic interaction between GBM and the stroma. Angiogenesis is another key feature of GBM, and vascular endothelial growth factor (VEGF), a mediator of *de novo* vessel formation in glioma (Plate et al., 1993), has been observed in reactive stromal astrocytes (Nagashima et al., 1999, 2002). These findings suggest another route by which astrocytes drive tumorigenicity.

### Secondary brain tumors

As mentioned, the dissemination of cancer cells from primary tumors to the brain is a common end-point for cancer patients with metastatic disease and is associated with poor prognosis. Most frequently, metastatic cells that colonize the CNS are derived from lung tumors, followed by melanoma, breast and renal malignancies (Barnholtz-Sloan et al., 2004). The molecular mechanisms governing such pathologies are beyond the scope of this article, and are yet to be fully characterized, although specific gene signatures have been shown to be predictive of brain metastasis (Bos et al., 2009; Harrell et al., 2012; Lee et al., 2012). To understand the complexities of the brain metastatic process, and the involvement of astrocytes, we must first consider the BBB.

The BBB serves as both a physical and metabolic barrier, separating the interstitial fluid of the brain parenchyma from peripheral blood flow, and is critical for maintenance of the neural environment in the CNS. It is comprised of multiple cell types, but is primarily regulated by the endothelium and astrocytes, with specialized tight junctions between endothelial cells serving to exclude metabolites. The unique composition of the BBB also serves as an obstacle to circulating cancer cell extravasation. Colonization of the brain parenchyma is not just reliant on traversing the endothelium, but also the glia limitans, the layer of astrocytic endfoot processes that provide structural and metabolic support to the endothelium.

Astrocytes contribute not just to BBB structure, by contacting the vascular endothelium, but also to its genesis, inducing tight junction formation between endothelial cells (Goldstein, 1988), and up-regulating expression of transporters such as the brain specific glucose transporter, GLUT1 (Boado and Pardridge, 1990; McAllister et al., 2001). Astrocytes also subserve a metabolic barrier function, for instance, inducing expression of endothelial cell enzymes such as manganese superoxide dismutase, the inducible metabolizer of oxygen free radicals (Schroeter et al., 2001). Calcium signaling between the endothelium and astrocytes (Leybaert et al., 1998; Braet et al., 2001) suggests that endothelial changes may also have a dynamic effect on astrocyte function. Hence, one could hypothesize that astrocytes will be activated as early as the initial steps of adhesion of metastasizing cells to the vascular endothelium.

Sophisticated imaging of fluorescent metastatic cells, using multi-photon laser scanning microscopy (MPLSM), has allowed the stages of brain metastasis to be visualized *in vivo* (Kienast et al., 2010). The tortuous nature of the cerebral micro-vasculature enforces a reduction in the speed of circulating cells and arrest at vascular branch points is observed. Such arrested cells can reside on the luminal side of the brain endothelium for up to 5 days, in contrast to elsewhere in the body where metastasizing cells rapidly extravasate within 24 h (Lorger and Felding-Habermann, 2010). Using MPLSM, extravasation into the perivascular space appeared to occur via active transmigration through mechanically induced pores in the endothelial membrane, as previously hypothesized (Kawaguchi et al., 1982). In addition, it was demonstrated that growth within the perivascular space is dependent on contact with the abluminal endothelial membrane (Carbonell et al., 2009), as has also been demonstrated in glioma (Winkler et al., 2009). Only upon growth of macrometastases, will the glia limitans be breached, allowing spread of metastases into the parenchyma (Saito et al., 2007).

The characteristic response of astrocytes to injury, seen in numerous neuropathologies, including glioma, has also been identified as a feature of the brain metastatic microenvironment. A wall of reactive astrocytes has been reported around haematogenous brain metastases in human post mortem tissue (Zhang and Olsson, 1995; He et al., 2006), and recently there has been great interest in establishing murine mouse models of brain metastasis to determine astrocyte reactivity *in vivo* (Mendes et al., 2005, 2007; Fitzgerald et al., 2008; Lorger and Felding-Habermann, 2010; Seike et al., 2011), as demonstrated in Figure 1. Astrocytic responses have been demonstrated in BALB/c mice in response to both the syngeneic 4T1 mammary carcinoma cell line, as well as in SCID mice in the response to the human MDA-MB-435 cell line, as early as 3 days post metastatic induction via intra-carotid inoculation (Lorger and Felding-Habermann, 2010). Astrocyte reactivity was induced whilst MDA-MB-435 cells were still intra-vascular, in contact with the luminal endothelial membrane, and continued throughout extravasation and growth over a 50 days time course. Likewise, Mendes et al. utilized a mammary carcinoma cell line, ENU1564, injected intra-cardially, to demonstrate astrogliosis in response to brain metastases in a rat model (Mendes et al., 2007).

Gliosis is not only found in response to metastases of mammary origin; Izraely et al. demonstrated astrocyte activation in nude mice in response to metastatic melanoma (Izraely et al., 2012) and Seike et al. demonstrated a wall of astrogliosis in nude mice in response to the human lung cancer cell, HARA-B. In this study, a positive correlation was found between tumor size and the extent of astrocyte activation and, interestingly, astrocyte activation was more robust in hippocampal metastases as compared to cortical metastases (Seike et al., 2011). Such differential astrocytic responses have also been observed in other disease models, such as intracerebral lipopolysaccharide (LPS) challenge (Espinosa-Oliva et al., 2011). These differential reactions may reflect the regional heterogeneity of astrocytes, both in terms of astrocytic density (which is greater in the hippocampus than the cortex), proliferation rates (which are higher in the hippocampal dentate gyrus than the cortex) (Emsley and Macklis, 2006) and molecular signature with regards to immune function. *In vitro*, hippocampal astrocytes have been shown to express higher levels of MHC Class II protein, Il-6 and ICAM-1 compared to cortical astrocytes, as well as displaying increased nitric oxide (NO) production (Morga et al., 1998), all of which one would hypothesize to modulate astrocyte response to metastatic growth. Indeed, studies of metastatic distribution in human patients, suggest that the hippocampus is a rare locale for tumor growth, as compared to the cerebellum or frontal lobe (Delattre et al., 1988; Ghia et al., 2007; Bender and Tome, 2011). Potentially, the more robust responses of astrocytes in the hippocampus, both on a cellular and immunological basis, could hamper metastatic growth.

As in glioma, the “double edged sword” of inflammation that is so often reviewed in the literature, not just in cancer (Hagemann et al., 2007; Lin, 2010; Rizzo et al., 2011) but in other diseases such as asthma (Balhara and Gounni, 2012), stroke (Doyle and Buckwalter, 2012), and neurodegeneration (Wyss-Coray and Mucke, 2002), appears to also be a feature of the astrocytic response to brain metastasis. *In vitro* studies have shown that astrocytes produce NO, via inducible nitric oxide synthase (Simmons and Murphy, 1992), and that this has tumoricidal effects in both primary and secondary cancer cell lines (Samdani et al., 2004). Evidence that NO induces GFAP upregulation (Brahmachari et al., 2006) suggests that this may result in increased detection of reactive astrocytes with disease progression.

However, a greater burden of evidence suggests a tumor promoting role for astrocytes. Immunohistochemical analysis of human biopsies has demonstrated up-regulation of endothelin [a cancer cell mitogen (Bagnato et al., 1997)] expression by tumor associated astrocytes (Zhang and Olsson, 1995). Moreover, co-culture experiments between astrocytes and various tumor cell lines indicate that astrocytes release soluble factors that can enhance tumor cell growth. For instance, endothelin-1, besides its role in vasoconstriction, has also been proposed to be mitogenic (Kasuya et al., 1994; Bagnato et al., 1997, 2002), and analysis of biopsy samples suggests that endothelin-1 is expressed by astrocytes in 85% of patient cases (Zhang and Olsson, 1995). Additionally, the receptor for endothelin-1, ET_B_, is upregulated in a brain-metastatic melanoma cell line over 3 fold, as compared to a non-metastatic cell line (Boukerche et al., 2004). This finding suggests that astrocytes may drive the molecular determinants of metastatic potential. Furthermore, incubation of the lung adenocarcinoma cell line PC14-PE6 with an immortalized astrocytic cell line induced cancer cell ERK1/2 phosphorylation (Langley et al., 2009), part of the MAP kinase signaling pathway heavily implicated in tumor progression. ERK1/2 phosphorylation has also been demonstrated in a metastatic mammary carcinoma cell line, ENU1564, in response to astrocyte conditioned media (Mendes et al., 2007). In this case, ERK1/2 activation was shown to increase tumor cell invasiveness *in vitro* via induction of MMP2 expression, as was seen in astrocyte-glioma co-culture models (Le et al., 2003).

Metastatic invasion may also be facilitated by astrocyte-derived heparanase, which degrades heparin sulphate proteoglycans, a major component of the extracellular matrix. Astrocyte heparanase expression has been demonstrated in the peri-infarct regions of *in vivo* stroke models (Takahashi et al., 2007; Li et al., 2012a) and in rat astrocyte-tumor cell culture models. In the latter case, it was shown that co-culture of astrocytes with brain-metastatic melanoma cell lines led to a super-additive increase in enzyme activity, potentially through neurotrophin signaling (Marchetti et al., 2000). Treatment of melanoma cell lines with astrocyte conditioned media led to increased cell invasion, an effect which was abrogated with antibody-mediated neutralization of heparanase. Further co-culture experiments between astrocytes and several lung cancer derived cell lines indicate that astrocytes secrete IL-6, TNF, and IL-1β, which stimulate tumor cell growth (Seike et al., 2011). It has also been suggested that astrocytes increase the anchorage-independent growth of cancer cell lines and that this correlates with metastatic ability *in vivo* (Fitzgerald et al., 2008), although the mechanism has yet to be identified.

Astrocytes have also been shown to induce transcriptional changes in co-cultured tumor cells that reflect the transcriptional changes seen *in vivo* (Park et al., 2011). Here, a Competitive Hybridization of Microarray Experiment (CHME) was used to tease apart genes upregulated in metastases as compared to the tumor micro-environment. In this case, human cancer cells were introduced into immuno-compromised mice, and differing gene signatures and methylation statuses between the cell populations were demonstrated. Subsequently, astrocytes were cultured with a breast carcinoma cell line, MDA-MB-231, and were shown to induce a similar genomic signature in the tumor cells to that seen *in vivo*, suggesting that astrocytes are mediators of tumor cell transcriptional reprogramming. For instance, brain metastatic cells were shown to upregulate genes involved in neuronal processes such as glutamate receptor signaling, axonal guidance, and neurotransmission. This novel approach to probing astrocytic function in brain metastasis demonstrates the key role astrocytes play in tumor progression, but also highlights the need to determine both the mechanism and the growth advantage conferred.

On balance, the studies detailed above suggest a pro-tumorigenic role for astrocytes, however, further *in vivo* studies are required to elucidate this. Besides these proposed roles in tumor pathogenesis, astrocytes have also been implicated in protecting tumor cells from chemotherapeutic agents. Co-culture of astrocytes with breast and lung cancer cell lines (MDA-MB-231 and PC14Br_4_, respectively) leads to up-regulation of survival genes such as *BCL2L1*, an anti-apoptotic member of the BCL-2 protein family (Kim et al., 2011). Such genes confer resistance to a range of chemotherapeutics, and are absent in cell lines at secondary sites other than the brain, highlighting a novel role for astrocytes. Further work has shown that the mechanism of cell protection is cell contact dependent and mediated via gap junction facilitated sequestration of calcium from tumor cells (Lin et al., 2010).

## Imaging astrocytes *in vivo*

The advent of molecular imaging, both as a stand-alone modality and combined with transgenic mouse models, has enabled the dynamic responses of astrocytes in numerous CNS pathologies to be visualized. Such techniques, in addition to yielding mechanistic insights, have potential for diagnostic imaging of neuroinflammation. *In vivo* research into the role of astrocytes in the tumor microenvironment is still in its infancy; however, using the numerous methodologies outlined below, there is much potential for better understanding the contribution of this most abundant CNS cell type to the pathogenesis of primary tumors and metastases.

### *In vivo* labeling of astrocytes

Astrocytes are primarily identified, *in vitro, in vivo*, and *ex vivo*, by their specific expression of GFAP, a cytoskeletal protein upregulated in astrogliosis, via antibody mediated detection methods, or transgenic systems, as outlined below. Alternatively, the propensity of astrocytes to specifically take up dyes such as sulforhodamine 101 (SR101), and facilitate their spread via gap-junctions (Nimmerjahn et al., 2004; Appaix et al., 2012), has enabled the study of astrocytic function, predominantly in slice models. Recently, such studies have been translated into the *in vivo* setting, with SR101 applied to the exposed cortex or injected intraperitoneally, immediately prior to imaging (Nimmerjahn and Helmchen, 2012). Using this technique, the intimate relationship between astrocytes and the endothelium in healthy tissue has been demonstrated, with gap-junction signaling between the two populations proposed (McCaslin et al., 2011).

Dyes, however, only enable transient labeling over a matter of hours and, therefore, techniques that allow long-term visualization are more useful for studying astrocytes in pathology. Viral vectors can be used to transfect cell types with fluorescent dyes. For example, a recombinant adenovirus-associated vector (AAV) has been used to stably and chronically transduce neurons and astrocytes, inducing green fluorescent protein (GFP) expression and allowing *in vivo* two photon microscopy of the visual cortex via cranial windows (Lowery et al., 2009). Additionally, BBB permeable, GFP-labeled anti-GFAP antibodies have very recently been used to image astrocytes, both *in vivo* and *ex vivo* (Li et al., 2012b). The potential for longitudinal imaging of astrogliosis by these means in response to CNS disease or injury will enable the temporal and spatial profile of reactivity to be mapped.

Transgenic models offer an even more stable approach. The specific expression of GFAP by astrocytes allows its promoter to be manipulated to drive the expression of reporter genes solely in astrocytes, as first described in a transgenic mouse model in which bacterial LacZ was placed under the GFAP promoter (Brenner et al., 1994). Since then, transgenic models have been engineered in which astrocytes express GFP (Zhuo et al., 1997; Nolte et al., 2001), allowing for the observation of astrogliosis in brain slices with fluorescent microscopy, and real-time *in vivo* detection of astrogliosis using multi-photon microscopy. This technique has been employed by several groups investigating systems such as retinal gliosis in diabetes (Kumar and Zhuo, 2010), retinal neurotoxicity (Ho et al., 2009) and NMDA-induced astrocyte activation (Serrano et al., 2008). This transgenic model can also facilitate sorting of astrocytes from surrounding brain tissue, allowing mRNA expression profiling in glioma (Katz et al., 2012), as described above.

Bioluminescence is another technique that allows visualization of reactive astrocytes *in vivo*, by placing the luciferase gene under the control of the GFAP promoter, as first described by Zhu et al. (2004). This transgenic mouse line has enabled astrocyte activation, in response to both glioma and metastatic cell lines implanted directly into the brain, to be studied dynamically and non-invasively (Lee et al., 2011). Using this approach, Lee et al. showed that astrogliosis peaks 3 days after tumor implantation and showed a biphasic time course over a 28 days experimental period in both glioma and metastatic models. An extended astrocyte response to brain metastasis has also been observed in other groups (Lorger and Felding-Habermann, 2010; Seike et al., 2011), including our own (unpublished observations), using conventional immunohistochemistry.

It is evident that labeling of astrocytes is highly dependent on GFAP. It should be noted however that not all astrocytes express detectable levels of GFAP and its expression in disease states can be variable (Wang and Walz, 2003). S100β, a calcium binding protein (Baudier et al., 1986), is often used as a astrocyte marker, however there is cross-reactivity with oligodendrocytes (Deloulme et al., 2004; Hachem et al., 2005). In addition, a number of other proteins are selectively expressed by astrocytes, such as the enzyme glutamine synthetase and aquaporin-4, but their sub-cellular distribution renders them impractical for astrocyte labeling (Yang et al., 2011). One should also note the differential expression of astrocytic markers throughout development. For instance, the glutamate transporter GLT-1 has been shown, *in vitro*, to be expressed at high levels in astrocyte cultures from embryonic stages, but not post-natal time points, whereas the glutamate transporter GLAST is highly expressed in astrocytes cultured from early post-natal time points, with a decline seen from p10 (Stanimirovic et al., 1999). *In vivo*, the spatial expression of these two transporters appears to change during post-natal development (Voutsinos-Porche et al., 2003).

Rather than pursuing global markers for astrocyte identification, attempts to identify markers for different phenotypes of astrocyte reactivity would allow greater understanding of the functional impact of gliosis at different spatial and temporal locales. For instance, the cytoskeletal protein vimentin, can be used to define astrocytes proximal to ischemic lesions, where neuronal damage is present, but is absent in distal gliosis (Petito et al., 1990; Wang and Walz, 2003). A recent comprehensive transcriptional profile of astrocytes in response to two different *in vivo* challenges in mice, LPS and middle cerebral artery occlusion (MCAO), demonstrates differential molecular profiles in response to the nature of the insult and time from lesion induction (Zamanian et al., 2012). For example, nestin and tenascin-C expression were observed in the astrocyte response to stroke, but not LPS-induced inflammation. Studies such as these demonstrate the considerable heterogeneity in astrocytic phenotypes, within and between disease states, and highlight the need for imaging agents that reflect this diversity of responses.

### Functional imaging

Astrocyte reactivity is just one facet of the contribution of glia to the healthy and diseased brain. Astrocyte *excitability* [which is based on oscillations in intracellular Ca^2+^ concentration ([Ca^2+^]_i_)] elicits effects in the healthy and diseased brain (Halassa et al., 2007; Kuchibhotla et al., 2009), and requires sophisticated imaging modalities to elucidate its functional impact. Neurotransmitter release by neurons leads to elevation of astrocyte [Ca^2+^]_i_ upon binding to receptors on peri-synaptic astrocytic membranes, and consequent activation of Phospholipase C. Spontaneous excitation can also occur. Such elevations in [Ca^2+^]_i_ result in astrocytic signaling to neurons, due to the release of gliotransmitters such as glutamate and D-serine (Araque et al., 2001; Fellin et al., 2004; Henneberger et al., 2010), and to other astrocytes.

Calcium oscillations and waves are typically investigated using chemical calcium indicators. Upon calcium chelation, the spectral properties of such dyes are altered such that they provide a fluorescent read-out for [Ca^2+^]_i_ (Tsien, 1988). A cell permeable acetoxymethyl (AM) ester form of the indicator dye is often used. Once inside the cell, the AM bond is cleaved by endogenous esterases and the dye is trapped within the cell. Using bulk loading techniques all cells will take up the dye. However, by using astrocyte marker dyes, such as SR101, astrocytes can be identified (Nimmerjahn et al., 2004). More recently, genetically encoded molecules have been developed (Miyawaki et al., 1997), which confer the advantage of being targetable to a specific cell type or subcellular compartment (Shigetomi et al., 2010). This approach is particularly exciting for *in vivo* imaging, as the stable expression of the calcium reporter allows for long term imaging of calcium signals (Mank et al., 2008). Generally these indicators comprise of a calcium binding protein fused to a variant of GFP. Upon calcium binding, a conformational change of the indicator results in either a change in the fluorescence resonance energy transfer (FRET) between the flanking GFPs (Miyawaki et al., 1997; Pologruto et al., 2004) or a fluorescence change in the molecule itself (Nagai et al., 2001; Nakai et al., 2001; Shigetomi et al., 2010).

Owing to the optical scattering properties of brain tissue, fluorescence imaging is degraded with increasing depth below the brain surface. Light scattering can be decreased by using infra-red-shifted light, compared to the use of visible wavelengths. As light of a longer wavelength has less energy per photon, the near-simultaneous absorption of multiple photons is required in order to excite a fluorophore, as occurs in multi-photon microscopy. Although most chemical calcium indicator dyes were originally developed for single photon microscopy, some (e.g., rhod-2, Fluo-4) also have sufficient two-photon cross-section.

Historically, two-photon imaging of calcium oscillations and waves in astrocytes has been limited to slice preparations (Mulligan and MacVicar, 2004; Tian et al., 2005; Di Castro et al., 2011). However, translation of the technique into *in vivo* systems has recently become possible. For example, Cirillo et al. have demonstrated calcium signaling in spinal cord astrocytes in response to sensory stimulation (Cirillo et al., 2012). Similarly, whisker stimulation was shown to elicit astrocyte responses in the barrel cortex (Tian et al., 2006; Wang et al., 2006). With regards to astrocyte calcium signaling in the diseased brain, elevated signaling has been observed in status epilepticus (Ding et al., 2007) and has also been demonstrated in the ischemic penumbra following stroke, where such increased signaling has been linked to increased neuronal damage (Ding et al., 2009). To date, application of calcium signaling imaging in CNS tumors, has been restricted to *in vitro* preparations of glioma cells (Charles et al., 1992; Yamasaki et al., 1994) or astrocyte and glioma cell co-cultures (Zhang et al., 1999). The latter, gap-junction dependent, interaction has been shown to be necessary for glioma invasion (Oliveira et al., 2005). Mediators of inflammation have also been shown to modulate astrocyte calcium signaling *in vitro* (Hamby et al., 2012) and, hence, one would anticipate alterations in signaling in the tumor microenvironment that could be probed *in vivo* using the novel methodologies outlined above.

### Magnetic resonance imaging

Magnetic resonance imaging (MRI) provides a powerful tool for imaging soft tissue contrast in the brain. As discussed, changes in astrocytic morphology accompany astrogliosis, and there have been attempts to image these changes with MRI. In both an Endothelin-1 induced ischemia rat model and an NMDA-induced model of cytotoxicity, T_1_ relaxation times have been shown to increase independent of changes in cerebral blood flow. The observed T_1_ hypointensities were seen in areas correlating to astrocyte activation and were reduced upon administration of arundic acid (Sibson et al., 2008), a selective inhibitor of astrocyte activation (Tateishi et al., 2002).

Other MRI parameters have been proposed to visualize astrocyte activation. Diffusion tensor imaging (DTI) facilitates observation of tissue microstructure, by both the rate and direction of water proton diffusion through a given area. By modeling diffusion information in a different manner, accounting for diffusion kurtosis (DK), it has been proposed that subtle heterogeneity of tissue can be further characterized. The work of Zhuo et al. suggests that changes in DK can be correlated with astrocyte activation and, thus, DK imaging could be used as a tool for investigating traumatic brain injury (Zhuo et al., 2012). Both this modality and the changes in T_1_ relaxation described above are still under review with regards to imaging tumor-induced gliosis. The resolution of MRI may not allow gliotic regions to be distinguished from the neoplasm itself, however, both of these techniques could hold potential as surrogate markers for *clinical* tumor detection.

Astrocytes are highly metabolically active, and multiple metabolic changes also accompany astrogliosis. For example, increased antioxidant activity is observed upon CNS insult; IL-1β signaling leads to the expression of cerruloplasmin, which buffers free copper ions and oxidizes ferrous iron (Kuhlow et al., 2003). Altered metabolic activity is a further feature of astrogliosis; in a ciliary neurotrophic factor (CNTF) induced model of astrogliosis, fatty acid oxidation and ketone body metabolism is increased, alongside decreased glycolysis (Escartin et al., 2007), conferring resistance to metabolic insults.

Under normal conditions, it has been proposed that the ATP requirements of astrocytes are primarily served by glycolysis (Pellerin and Magistretti, 1994, 1997), with the end-product, lactate, extruded into the extracellular space either for uptake by neurons (Aubert et al., 2005) or clearance from the brain. However, the TCA cycle and subsequent oxidative phosphorylation are also a source of ATP (Serres et al., 2008), and recent transcriptome analyses of acutely isolated mouse cortical astrocytes have demonstrated elevated levels of TCA cycle enzymes as compared to neurons, combined with high mitochondrial numbers in astrocyte foot processes (Lovatt et al., 2007). Whilst, to date, metabolic changes in reactive astrocytes surrounding CNS tumors have not been investigated, studies demonstrating increased glucose utilization by astrocytes in response to TNF, IL-1β, IL-6, and IFN-γ (Yu et al., 1995; Gavillet et al., 2008; Belanger et al., 2011), and the synergic activity of TNF and IL-1β on astrocyte metabolic activity (Gavillet et al., 2008), suggest that astrocytes in the pro-inflammatory microenvironment will be in a metabolically hyperactive state. Additionally, the ability of astrocytes to protect tissue during oxidative stress via the secretion of thiols and enhanced glutamate uptake can be modulated by the cytokine context (Garg et al., 2009). If this is indeed the case, a metabolic marker of astrocyte activity, *in vivo*, could be of considerable use.

Specificity for astrocyte metabolism, as opposed to neuronal activity, can be achieved through the differential uptake of certain metabolic substrates. In particular, acetate is preferentially taken up by astrocytes over neurons (Waniewski and Martin, 1998) and incorporated into the TCA cycle. ^13^C magnetic resonance spectroscopy (MRS) studies have demonstrated that it is possible to detect the transfer of ^13^C label from [2-^13^C]acetate into glutamine within astrocytes. The ^13^C label is first transferred to glutamate via the TCA cycle and exchange between the TCA cycle intermediate α-ketoglutarate (α-KG) and glutamate (Kanamatsu and Tsukada, 1999; Sibson et al., 2001). Both the α-KG and glutamate pools in astrocytes are at an extremely low concentration and, thus, are undetectable by the relatively low sensitivity ^13^C MRS. Subsequently, however, the labeled glutamate is converted to glutamine, which is a much larger metabolic pool (*ca*. 5–6 mM) and label flux into this pool is detectable by ^13^C MRS (Sibson et al., 1997). The flux of ^13^C label from acetate to glutamine can be converted into absolute rates of astrocytic TCA cycle (oxidative metabolism) through the use of metabolic modeling (for review see de Graaf et al., 2011). This ^13^C MRS approach has been used in transgenic mouse models of Alexander disease to show astrocyte dysfunction, with regards to reduced utilization of acetate for the synthesis of glutamate and glutamine (Meisingset et al., 2010), as well as in human studies. For instance, human studies in patients with Alzheimer's disease indicate that [2-^13^C]acetate can be used to detect gliosis (Sailasuta et al., 2011), with enhanced metabolism of acetate to ^13^C bicarbonate detected. While MRS has yet to be used to probe stromal astrocyte metabolism, studies of neoplastic astrocytes in glioma suggest that intermediates detected with spectroscopy (Doblas et al., 2012), could be translated into useful imaging tools in PET. Of relevance here is the use of the PET tracer ^18^F-acetate which has been used to demonstrate enhanced glial metabolism in both a rat stroke model and a glioblastoma model (Marik et al., 2009). In humans, the PET tracer ^11^C-acetate has been used for the detection of gliomas (Liu et al., 2006; Tsuchida et al., 2008). The use of nuclear imaging to detect gliosis will be further discussed below.

### SPECT and PET imaging

Nuclear imaging, with single photon emission computed tomography (SPECT) and positron emission tomography (PET), is a tool routinely used in the diagnosis of cancer. Radiolabeled tracers, primarily ^18^F-deoxyglucose (FDG), are used to detect tumor growth based on their hyper-metabolism of glucose as compared to healthy tissue. If astrocytes in and around tumors are truly hyper-metabolic (see above) then it is likely that FDG tumor detection may reflect, in part, reactive astrocytosis. However, imaging the tumors themselves is not the only possibility for the detection of neoplasms. The use of radiolabeled compounds to bind to astrocyte specific markers, or indirect measures of astrocyte activity, are avenues that have been pursued in many CNS diseases, with some agents being translated into a clinical setting. Whilst SPECT and PET cannot provide sufficient spatial resolution for probing individual cells, or sub-populations of cells, these approaches do allow detection of neuroinflammation, which will facilitate disease diagnosis and monitoring.

^99^Tc-HMPAO is a BBB-permeable SPECT imaging agent, with non-specific uptake by the brain proportional to blood flow. Retention of the compound is dependent on its intracellular reduction and, thus, the high glutathione concentration present in astrocytes leads to increased and sustained uptake of ^99^Tc-HMPAO by astrocytes as compared to neurons (Slosman et al., 2001). Although not specific for astrocyte activation, retention of this compound is enhanced in gliotic lesions in disease states such as dementia and Alzheimer's disease (Slosman et al., 2001) and herpes encephalitis (Launes et al., 1995). Alternatively, ^11^C-deuterium-L-deprenyl (^11^C-DED) is an irreversible monoamine oxidase B inhibitor. Activated astrocytes express elevated levels of monoamine oxidase and, consequently, this agent can be used to detect astrogliosis with PET imaging, as has been demonstrated to date in pathologies such as Alzheimer's (Carter et al., 2012), amyloid lateral sclerosis (ALS) (Johansson et al., 2007), and Creutzfield–Jakob disease (Engler et al., 2012).

**Figure 2:**
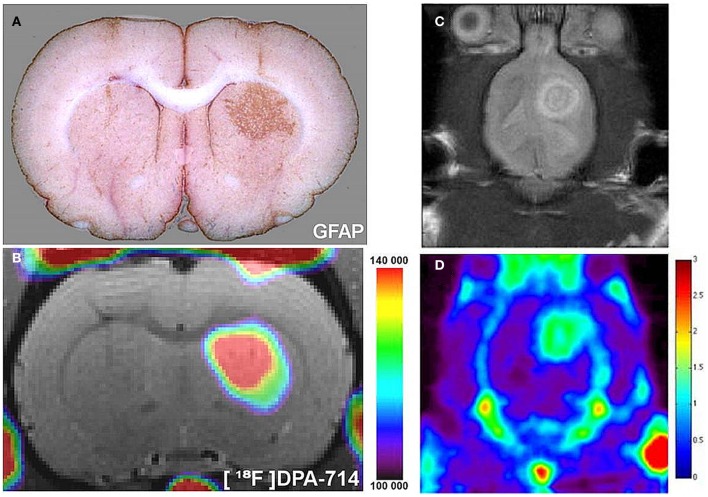
**Radiolabeled TSPO ligands can be used to image astrocyte activation *in vivo*. (A)** In a rat model, astrocytes were chronically activated (GFAP staining, brown) by lentiviral gene transfer of the cytokine ciliary neurotrophic factor (CNTF). **(B)** ^18^F-labeled DPA-714, a TSPO ligand, binding spatially correlates with areas of astrocyte activation, as observed with *in vivo* PET imaging. As adapted from Lavisse et al. (2012). Such TSPO targeted agents can be used to image TSPO expressing gliomas in pre-clinical models. **(C)** A pre-clinical rat model of glioma detected with MRI **(D)** ^18^F-PBR06, a TSPO ligand, binding spatially correlates with glioma growth, as observed *in vivo* with PET. As adapted from Buck et al. (2011).

Probing the increased metabolic activity evident in gliosis at the mitochondrial level has enabled *in vivo* imaging of astrocytes in multiple CNS pathologies, and is a promising tool for monitoring primary and secondary tumor growth. Translocator protein (TSPO—also known as the peripheral benzodiazepine receptor) is an outer mitochondrial membrane protein (Squires and Brastrup, 1977), and has multiple functions, including cholesterol import for steroid synthesis (Hauet et al., 2005), regulation of mitochondrial metabolism (Hirsch et al., 1989), and apoptosis (Hirsch et al., 1998). These diverse functions render knock-out models embryonically lethal (Papadopoulos et al., 1997). Although the TSPO was originally thought to be exclusively upregulated on microglia in disease states, recent studies have also demonstrated increased expression on astrocytes in humans, as well as in rodent models (Cosenza-Nashat et al., 2009; Lavisse et al., 2012), as shown in Figure 2. Consequently, interest has grown in the development and use of radiolabeled compounds against TSPO for imaging neuroinflammation in diverse CNS pathologies, such as multiple sclerosis (Harberts et al., 2012), dementia (Cagnin et al., 2001), Alzheimer's disease (Versijpt et al., 2003) and stroke (Gulyas et al., 2012a,b), as reviewed in (Ching et al., 2012).

In the context of imaging astrocytes in primary brain tumors (Figure 2), numerous glioma cell lines have been shown to highly express TSPO (Winkeler et al., 2012), and recent studies in rat models of glioma (Buck et al., 2011; Tang et al., 2012; Winkeler et al., 2012) suggest that this approach could be used as a clinical tool to detect brain tumors. Work in our own group suggests that radiolabeled anti-TSPO agents can also be used to detect brain metastases, on the basis of the large area of astrogliosis associated with these tumors (unpublished work). As discussed with regard to MRI, these techniques are unlikely to allow differentiation of tumor growth from associated astrogliosis, especially as TSPO upregulation has been observed in metastatic breast tumors (Zheng et al., 2011; Batarseh et al., 2012). However, this approach does hold potential as a surrogate biomarker for the clinical detection of CNS tumors.

## Conclusions

The role of the microenvironment in tumor progression has been extensively reported, and an inflammatory response to both primary and secondary brain tumors is becoming increasingly apparent. As detailed above, astrocytes are key players in the CNS response to tumor growth, and there have been a flurry of studies attempting to uncover the role of these glial cells in disease progression. Many of these studies were conducted *in vitro*, and suggest both pro- and anti-tumor effects. However, the balance of evidence seems to point toward a pro-tumorigenic role for astrogliosis, with the release of growth factors and matrix remodeling enzymes. Now that more sophisticated animal models have been developed, we can begin to understand the complex role of these cells *in vivo*. Importantly, functional imaging measurements, via methods such as calcium signaling, will allow a greater understanding of how astrocytes communicate in the tumor microenvironment. Multinuclear MRS will yield insights into tumor metabolism, and potentially elucidate the metabolic interactions between astrocytes and tumor cells. To elucidate astrogliotic functions in response to tumor growth, numerous imaging modalities can be utilized. Not only do these techniques allow the spatial and temporal profile of gliosis to be visualized and quantified, but they may potentially yield mechanistic insights through functional imaging and new clinically-relevant diagnostic approaches.

### Conflict of interest statement

The authors declare that the research was conducted in the absence of any commercial or financial relationships that could be construed as a potential conflict of interest.
